# Aquatic Ferrous Solutions of Prebiotic Mineral Salts as Strong UV Protectants and Possible Loci of Life Origin

**DOI:** 10.1089/ast.2023.0011

**Published:** 2023-07-10

**Authors:** Vladimir Subbotin, Gennady Fiksel

**Affiliations:** ^1^ Department of Human Oncology, University of Wisconsin, Madison, Wisconsin, USA.; ^2^ Department of Nuclear Engineering and Radiological Sciences, University of Michigan, Ann Arbor, Michigan, USA.

**Keywords:** Ferrous mineral salts, UV protectants, Origin of life

## Abstract

Liposomes are lipid-bilayer vesicles that spontaneously self-assemble from fatty acids (or other amphiphiles) in water by encapsulating surrounding aqueous media. After British scientist Alec Bangham described this phenomenon in the early 1960s, they became a prominent participant in the hypotheses on life origin, particularly in the Lipid World model. A novel scenario of self-sustained Darwinian liposome evolution is based on ever-present natural phenomena of cyclic day/night solar UV radiation and gravitational submersion of liposomes in the Archean aqueous media. One of the assumptions of the hypothesis is the UV-shielding ability of the Archean waters that could protect the submerged liposomes from the damaging solar UV radiation. To corroborate the idea, we measured UV absorption in aquatic solutions of several ferrous mineral salts assumed to be present in Archean pools. Single-agent solutions of simple salts such as FeCl_2_—iron dichloride, FeCl_3_—iron trichoride, Fe(NO_3_)_3_—ferric nitride, NH_4_Fe(SO_4_)_2_—ferric ammonium sulfate, and (NH_4_)_5_[Fe(C_6_H_4_O_7_)_2_]—ferric ammonium citrate were tested. These direct measurements of UV light absorption supplement and reinforce the proposed hypothesis.

## 1. Introduction

This article is a follow-up to our previous article (Subbotin and Fiksel, 2023) in which a novel scenario of self-sustained Darwinian evolution of the liposomes driven by ever-present natural phenomena of solar UV radiation, day/night cycle, gravity, and the formation of liposomes in the Archean aqueous media was proposed. One of the central and critical assumptions of the hypothesis is the UV-shielding ability of the Archean waters to protect the submerged liposomes from the damaging solar UV radiation. An important step to corroborate the idea is to determine whether the presumed composition of Archean waters could provide such a UV shielding.

The analysis of UV attenuation by media mimicking the Archean ocean has a century-long history, including the UV signal registration by photography (Hodgman, 1933), by spectrophotometry (Quickenden and Irvin, 1980; Cleaves and Miller, 1998), and by effects on biological targets: preservation of nucleosides (Todd *et al.,* 2021) and DNA (González-Ramírez *et al.,* 2023); survival of algae (Gómez *et al.,* 2007; Wang *et al.,* 2009) and bacteria (Gauger *et al.,* 2015; Mloszewska *et al.,* 2018).

Some studies (Mulkidjanian *et al.,* 2003) have indicated that water-soluble nitrogenous bases (adenine, thymine, uracil, cytosine, and guanine) could provide strong UV shielding. In addition to various biological compounds, it would be logical to explore the effect of simple aqueous solutions of mineral salts typical for marine water. Recent fundamental analysis of UV transmission in prebiotic waters (Ranjan *et al.,* 2022) suggests that ferrous waters are strong UV absorbers. Similar results were obtained in a study of UV absorption in martian brines (Godin *et al.,* 2020). Those results are aligned with previous experimental work on UV shielding utilizing either instrumental (Cleaves and Miller, 1998) or biological detection systems (Gómez *et al.,* 2007).

While the strong UV shielding by ferrous waters appears unfavorable to the UV-origin-of-life scenarios (Ranjan *et al.,* 2022), it would be highly advantageous for UV protection of amphiphiles' assembles explored in our scenario (Subbotin and Fiksel, 2023). Following the previous reports on UV-shielded properties of ferrous waters (Godin *et al.,* 2020; Ranjan *et al.,* 2022), we tested the UV-shielding properties of simple and complex salts (FeCl_2_—iron dichloride, FeCl_3_—iron trichoride, Fe(NO_3_)_3_—ferric nitride, NH_4_Fe(SO_4_)_2_—ferric ammonium sulfate, and (NH_4_)_5_[Fe(C_6_H_4_O_7_)_2_]—ferric ammonium citrate) that could be present in the Archean waters (Miller and Urey, 1959; Handschuh and Orgel, 1973; Keefe and Miller, 1996; Gómez *et al.,* 2007; Breslow *et al.,* 2010; Hardy *et al.,* 2015; Patel *et al.,* 2015; Sproul, 2015; Kim *et al.,* 2016).

The present study was intended to collect results on several promising UV absorbers using the same methodology in a single publication. A difference from the previously published research on this subject is that the absorbance measurements were wavelength-integrated within a spectral range of 200–400 nm, which we considered potentially most damaging. One of the outcomes of this work is the development of an experimental platform to experimentally measure the survival and destruction of liposomes under intense UV radiation in different mineral salt solutions. This part of the study is a work in progress.

## 2. Materials and Methods

### 2.1. UV source and detector

**FIG. 1.**
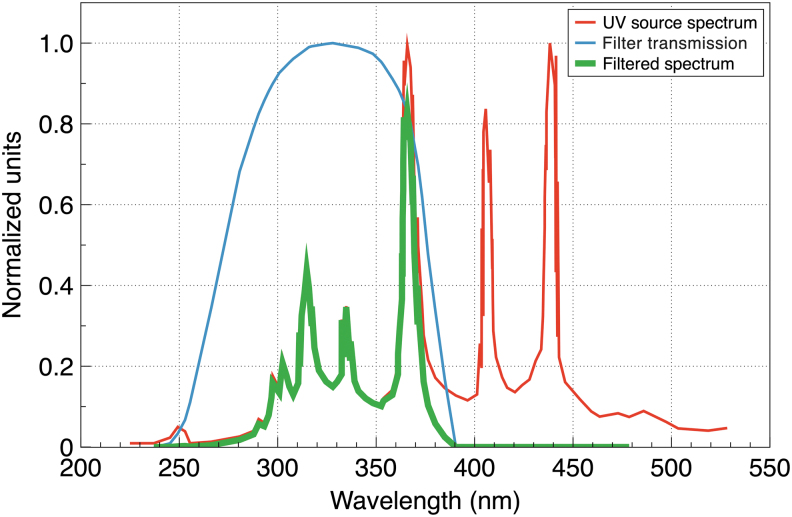
Spectra of the UV source (red), filter transmission (blue), and filtered UV radiation (green). Spectra of the Zeiss HBO 50 W illuminator from Crowther (2021), with permission from the author; spectra of the filter transmission from the manufacturer's website http://www.tangsinuo.com.

In the present study, single-agent solutions of the above salts were tested for UV-shielding properties using direct UV detection by a UV light sensor and a mercury bulb as a UV source. We used a mercury 50 W bulb mounted in the Zeiss HBO 50 W illuminator as a UV source. The bulb and the illuminator were parts of an LSM 501 microscope (Zeiss, Germany, manufactured in 1997). The spectral power of the radiation has multiple peaks in a UV range from 200 to 400 nm as well as in the visible part of the spectrum—Fig. 1. The visible light energy is neither sufficient for the synthesis nor decomposition of the liposomes. However, since the UV sensor we used is somewhat sensitive to the visible component of the mercury lamp, that part of the spectrum was filtered out in order not to interfere with the measurements. To filter out the visible region, we used a ZWB1 UV bandpass filter (Shijiazhuang Tangsinuo Optoelectronic Technology Co., China). Figure 1 shows the full UV source spectrum (red), the filter transmission spectrum (blue), and the spectrum of the filtered radiation (green), which is contained between 250 and 390 nm with a power-averaged wavelength of about 330 nm. The UV light was registered by a UV Light Sensor #28091 (Parallax Inc., Rocklin, CA) with a spectral UV sensitivity between 200 and 400 nm.

### 2.2. UV-attenuating media

We tested the following salts: FeCl_2_—iron dichloride, FeCl_3_—iron trichoride, Fe(NO_3_)_3_—ferric nitride, NH_4_Fe(SO_4_)_2_—ferric ammonium sulfate, and (NH_4_)_5_[Fe(C_6_H_4_O_7_)_2_]—ferric ammonium citrate, assumed to be present in Archean waters (Miller and Urey, 1959; Handschuh and Orgel, 1973; Keefe and Miller, 1996; Gómez *et al.,* 2007; Breslow *et al.,* 2010; Hardy *et al.,* 2015; Patel *et al.,* 2015; Sproul, 2015; Kim *et al.,* 2016).

### 2.3. Test tube

Each solution was placed into a glass tubing with a diameter of 19 mm. The bottom of the tubing was made of UV-grade fused quartz with a spectral transparency of 190–2500 nm (Alpha Nanotech Inc., Canada). A quartz condenser lens focuses the UV light at a size of about 10 × 10 mm at the solution's surface at approximately 100 mm^2^. The UV detector was positioned underneath the quartz bottom to measure the intensity of UV passing through the solution. In some tests, the tube's wall was lined with a UV-absorbing material to investigate the effect of UV reflection, which was found to be insignificant.

**FIG. 2.**
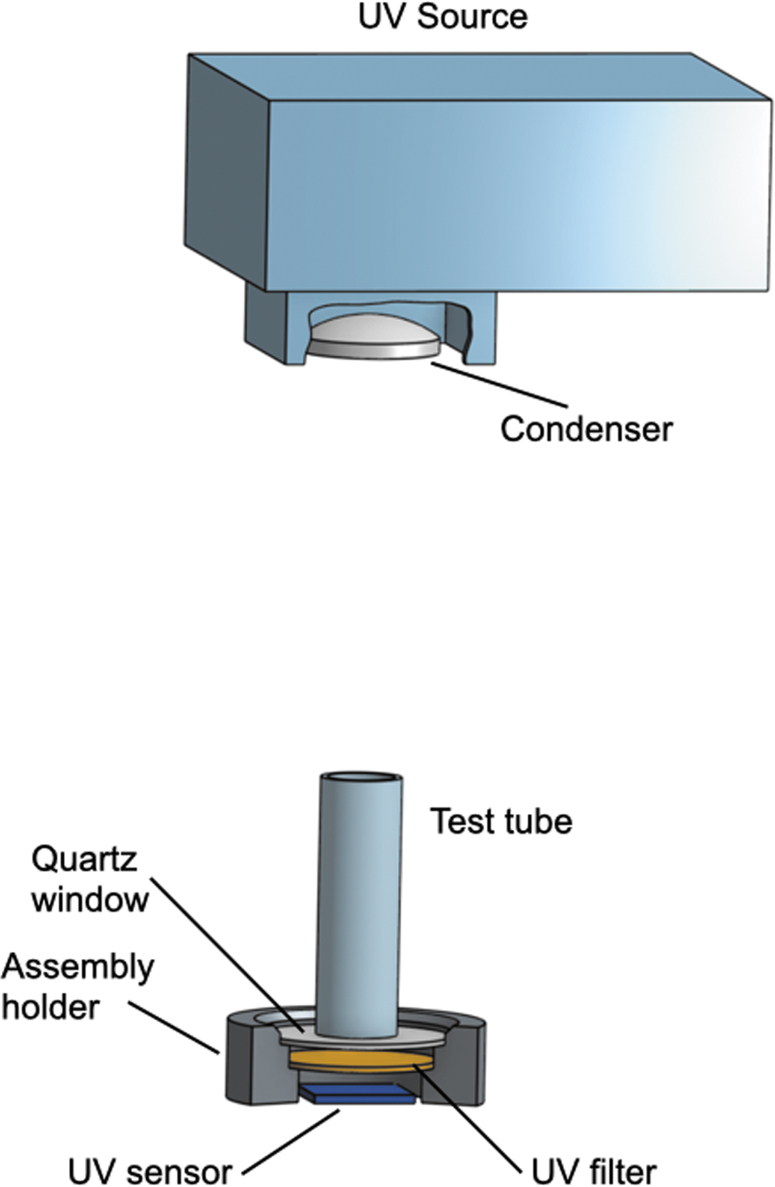
Experimental setup.

A sketch of the experimental setup is shown in Fig. 2. Full photos of the experiment can be seen in the Supplementary Material.

### 2.4. Experimental procedure and data analysis

Radiation transmission in matter is typically described by exponential attenuation with the absorbance $A=\log_{10}\left(I_{0}∕I\right)$, which is linearly dependent on the optical path length *d* and the density of the absorbent *n*.

(1) $$A=\log_{10}\left(I_{0}∕I\right)=n\sigma d∕\log\left(10\right)$$

Here *I*_0_ and *I* are, respectively, the incident and transmitted light intensities, and σ is the absorption cross section. In general, σ is radiation-wavelength dependent. Because our measurements are not spectrally resolved, the results should be considered as averaged over the 250–400 nm spectral interval.

Equation 1 depicts a linear dependence of the absorbance on the concentration, so after finding the slope of the linear regression $s=\sigma d∕\log\left(10\right)$, the cross section σ can be calculated from

(2) $$\sigma =\left(s∕d\right)\log\left(10\right)$$

Salt solutions with a mass concentration ρ ranging from 0 g/L (distilled water) to 2.5 g/L were prepared. The absorbent density *n* was determined from $n=N_{A}\rho ∕M=N_{A}m$, where $N_{A}$ is the Avogadro constant, *M* is the molar mass, and *m* is the molar concentration. The transmitted light intensity was measured by varying the absorbent density *n* at a fixed light pass *d*. The solutions were infused into the test tube up to a certain height, and the dependence of the transmitted UV intensity on the solution concentration was measured.

### 2.5. Results

**FIG. 3.**
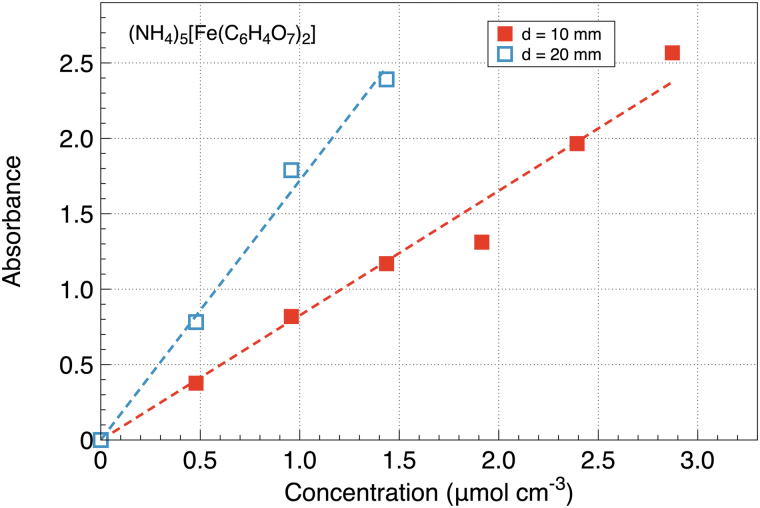
Dependence of UV absorbance on the concentration of ferric ammonium citrate taken at two light paths d= 10 and 20 mm. Symbols show the data points, and the dashed lines show linear fits.

All the absorbance plots show a similar linear dependence on the solution concentration in agreement with Eq. 1. For example, UV absorbance by ferric ammonium citrate (NH_4_)_5_[Fe(C_6_H_4_O_7_)_2_] taken at two light paths d=10 and 20 mm is shown in Fig. 3.

**FIG. 4.**
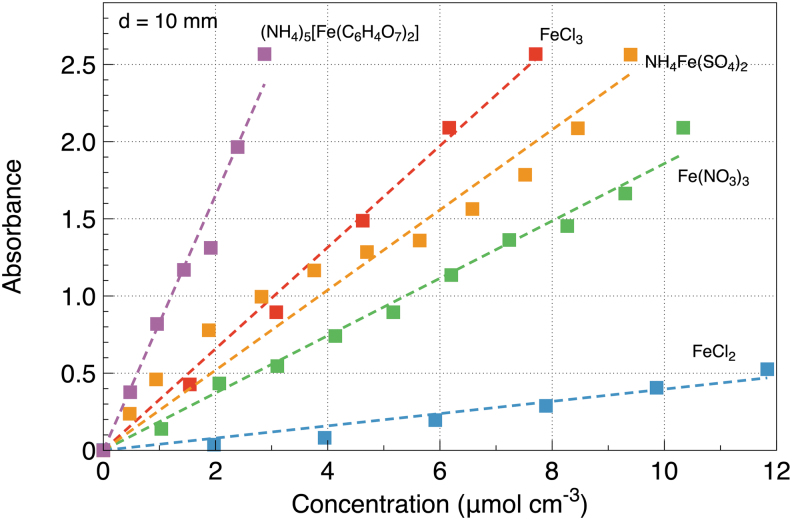
Dependence of UV absorbance on the concentration of all the tested salts at a light path of d= 10 mm. Symbols show the data points, and the dashed lines show linear fits.

Figure 4 shows data assembled over all the tested salts at a light path of d=10 mm.

The inferred absorption cross sections are shown in Table 1. The uncertainties are calculated from the statistical scatter of the data taken at different light paths as well as from the measurement accuracy of the experimental apparatus, such as the voltmeter, weight scale, and so on.

**Table 1.** UV Absorption Cross Sections for the Tested Salts and Submersion Depth to Achieve a UV Attenuation of 100 (*A* = 2) and 1000 (*A* = 3) at a Salt Mass Concentration of 2.5 g/L

Salt		(NH_4_)_5_[Fe(C_6_H_4_O_7_)_2_]	FeCl_3_	NH_4_Fe(SO_4_)_2_	Fe(NO_3_)_3_	FeCl_2_
σ (10^−19^ cm^2^)		32.3 ± 0.65	11.9 ± 0.70	11.3 ± 1.2	6.85 ± 0.35	1.63 ± 0.11
Depth (mm)	*A* = 2	4.9	4.1	7.2	11.1	23.9
	*A* = 3	7.4	6.3	10.8	16.6	35.8

It follows from the gravity-friction balance model (Subbotin and Fiksel, 2023) that at a typical liposome vesicle radius of 1 μm, the 12 h nighttime submergence depth is about 1–10 mm, depending on the specific gravity of the vesicle. Thus, the absorption should be high enough to provide a necessary UV attenuation at these submergence depths.

One of the primary soluble salts in Archean waters was likely FeCl_3_ at a suggested concentration of 2.5 g/L (Gómez *et al.,* 2007). Table 1 lists the submersion depth necessary to attenuate UV by a factor of 100 (*A* = 2) and 1000 (*A* = 3) at that concentration. We see that at least two salts, (NH_4_)_5_[Fe(C_6_H_4_O_7_)_2_] and FeCl_3_ provide an attenuation by a factor of 1000 within the 10 mm limit. The other salts are weaker UV attenuators and might not be able to provide the necessary protection.

## 3. Summary and Conclusions

Previously we offered a theoretical solution for the puzzle of temporal and spatial incidental co-occurrence of the molecules and assemblies necessary for life origin but which are products of unrelated physical/chemical events (Subbotin and Fiksel, 2023). Elaborating on the thoughts of Rutten and Oparin (Rutten, 1971; Oparin, 1974), we considered the water-air interface as a vital and crucial locus for the origin of life. It is where the assemblies of amphiphiles, synthesized in primordial waters, congregate and form liposomes. Likewise, the water-air interface is the plane to which the prebiotic molecules synthesized in the atmosphere (Miller, 1953; Miller and Urey, 1959) descend and where they can be entrapped during the liposomogenesis.

To overcome the inevitable destruction of the liposomes at the water-air interface by solar UV radiation, we hypothesized that the liposomes could entrap heavy solutes and then submerge deep into the water and thus become protected from UV damage. Because of the cyclical day-night nature of the UV radiation, the submerge velocity must be high enough to descend to a sufficient depth during the nighttime so as not to be subjected to the destructive UV during the following daytime. It follows from the gravity-friction balance model (Subbotin and Fiksel, 2023) that at a typical liposome vesicle radius of 1 μm, the 12 h nighttime submergence depth is about 1–10 mm, depending on the specific gravity of the vesicle. Thus, the absorption should be high enough to provide a necessary UV attenuation at these submergence depths.

Our measurements clearly demonstrate that for two tested salts, (NH_4_)_5_[Fe(C_6_H_4_O_7_)_2_] and FeCl_3_, at a concentration of 2.5 g/L, the UV intensity drops by a factor of 100 at a submersion depth of about 4.9 and 4.1 mm, respectively, and by a factor of 1000 at a submersion depth of about 7.4 and 6.3 mm, respectively, which falls within the range predicted by the model. For some types of minerals, particularly FeCl_3_, that concentration was considered typical for primordial waters on Earth (Gómez *et al.,* 2007; González-Ramírez *et al.,* 2023). While the Archean ocean might not have had millimolar ammonium levels, we extended the concentration range of the ammonium salt to compare all the minerals at similar concentrations in the lab. Further comparison, considering more realistic concentrations and associated effects of UV absorption, will be realized in future work. One promising conclusion drawn from our results is that shallow ferrous aqueous ponds (Mulkidjanian, 2011; Damer and Deamer, 2015; Deamer, 2017) located, for example, over iron-rich soils, could be a prominent candidate for a place of life origin.

## Credit Statements

Vladimir Subbotin: initial conceptualization, assembling experimental setup and experiments. Writing original draft, review, and editing.

Gennady Fiksel: elaboration of the hypothesis, methodology, modeling, validation, and analysis. Writing original draft, review, and editing.

## Supplementary Material

Supplemental data
