# Supplementary material for: Aquatic Ferrous Solutions of Prebiotic Mineral Salts as Strong UV Protectants and Possible Loci of Life Origin
Source: Astrobiology. 2023 Jul 10;23(7):741–5. doi: 10.1089/ast.2023.0011 (PMC10354304; doi:10.1089/ast.2023.0011)
Supplement: Supplemental data [file Suppl_Data.pdf]

Supplemental materials

Aquatic ferrous solutions of prebiotic mineral salts as strong UV protectants and possible loci of life origin

Vladimir Subbotin
University of Wisconsin, Department of Human Oncology

Gennady Fiksel
University of Michigan, Department of Nuclear Engineering & Radiological Sciences

UV source

We used a mercury 50W bulb mounted in the Zeiss HBO 50 W illuminator as a UV source. The bulb and the bulb housing were parts of an LSM 501 microscope (Zeiss, Germany, manufactured in 1997). The original power source (ARC Lamp power supply, Carl Zeiss) was used. A heavy stand from a stereomicroscope was used to secure HBO 50 W illuminator and align the UV beam (Figure S1).

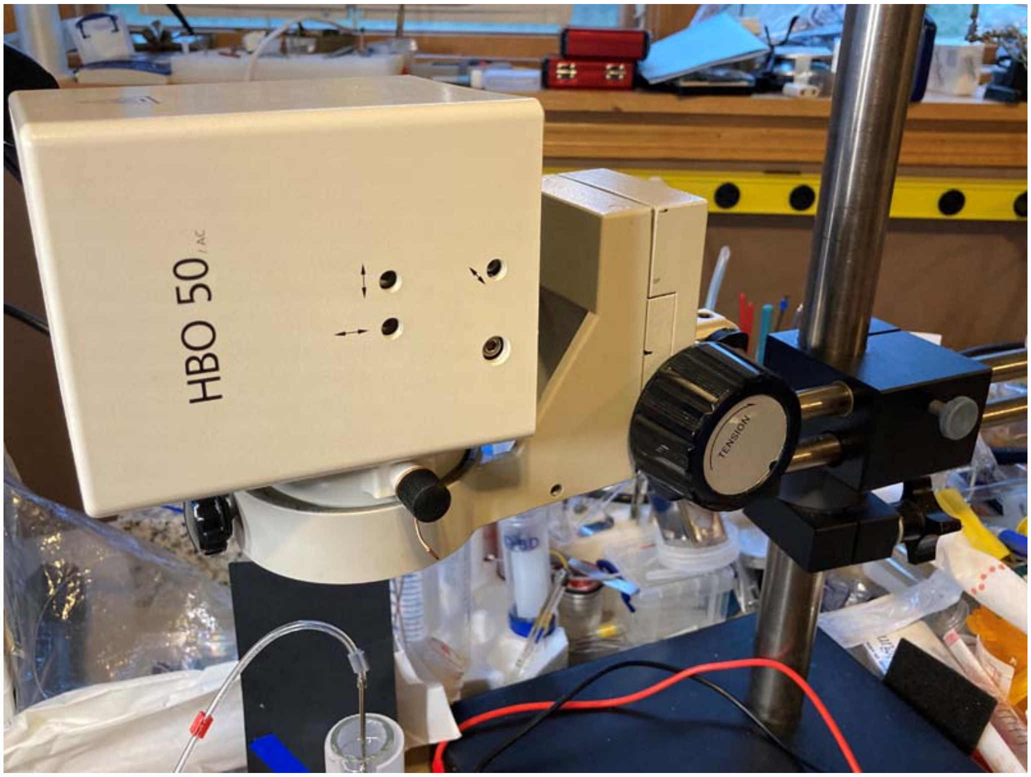

Figure S1.

Test tube

The test tube was made from a 100 mm length, 19 mm ID glass COE 33 Boro tubing. The bottom of the tubing was made of UV-grade fused quartz plate with a spectral transparency

of 190-2500 nm (Alpha Nanotech Inc., Canada). The quartz plate was glued to the glass tube using 100% silicone aquarium sealant (Figure S2).

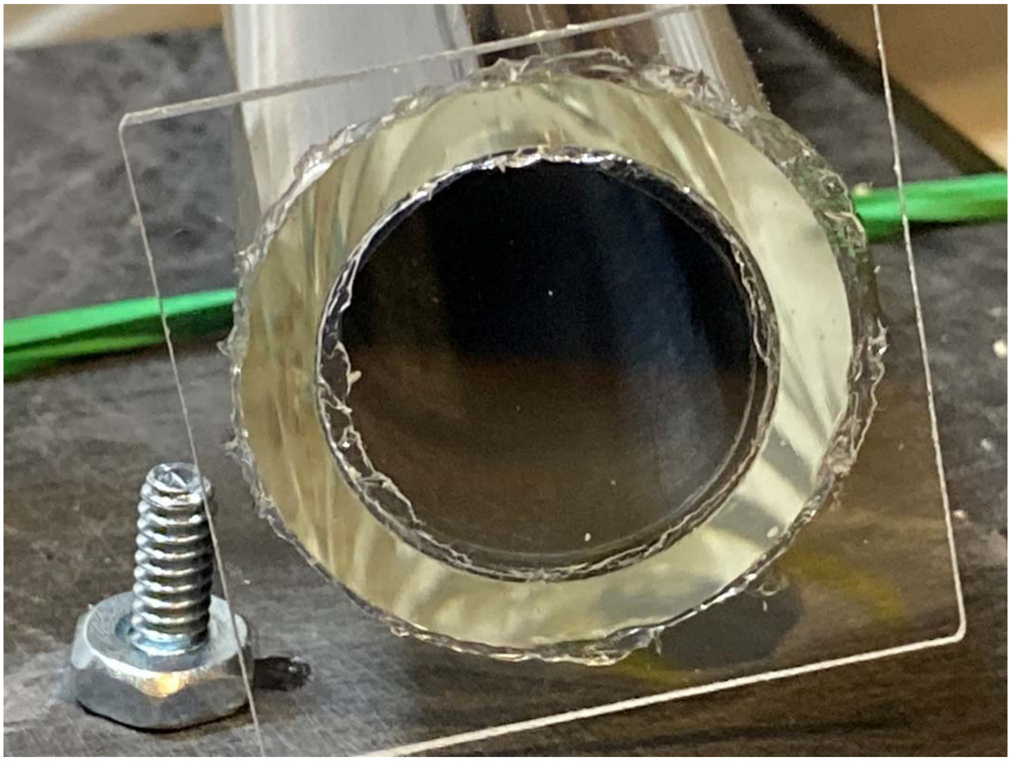

Figure S2.

To avoid fluid retention and bubble formation on the test tube's wall during the tube filling with solutions, it was done with a 14 G stainless steel delivery tube lowered to the quartz bottom. The delivery needle was secured and close to a wall inside the test tube with stainless wire rings. In initial experiments, the tube's wall was lined with a UV-absorbing material to investigate the effect of UV reflection (Figure S3).

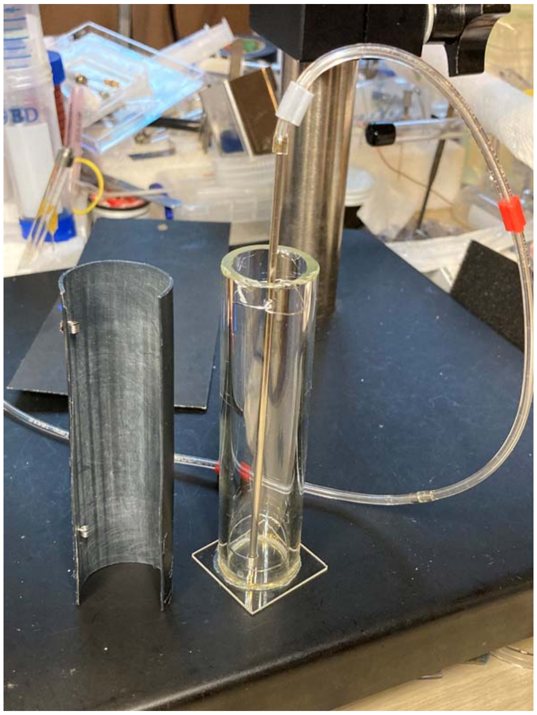

Figure S3.

UV detector
The UV Light Sensor #28091 (Parallax Inc., Rocklin, CA) was mounted inside a light-tight plastic enclosure. The ZWB1 UV bandpass filter (Shijiazhuang Tangsinuo Optoelectronic Technology Co., China) was placed inside a plastic housing and secured on top of the UV sensor (Figure S4).

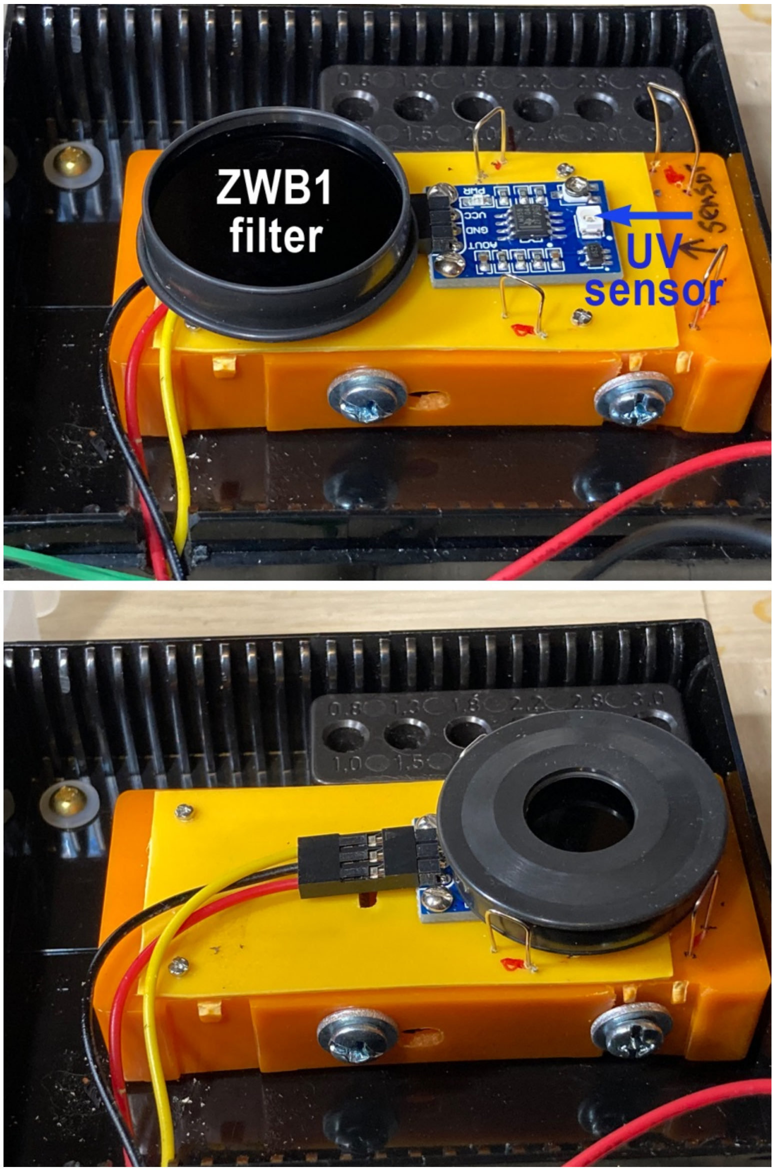

Figure S4.

Experimental setup.
Salts solutions were infused into the test tube either manually or with the Harvard Apparatus syringe pump (Figure S5).

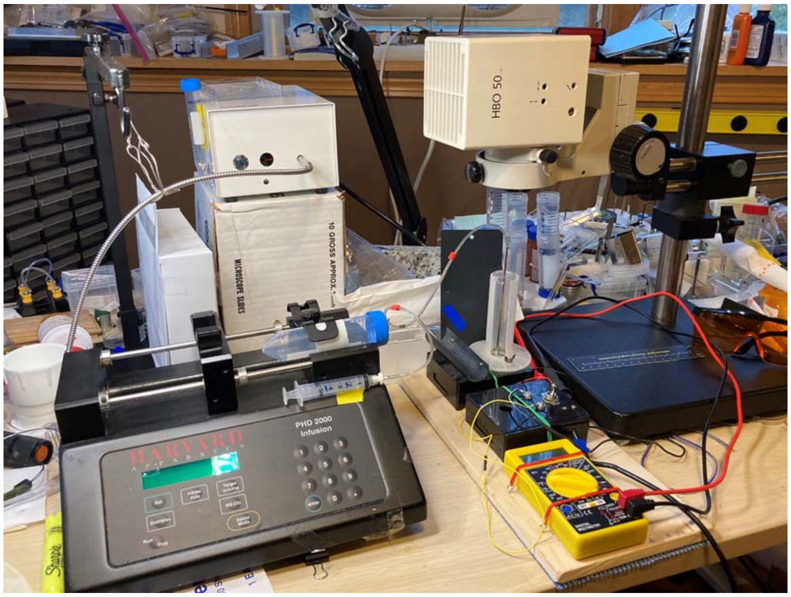

Figure S5.

Please add:
A – UV light off,
B – UV light on

The test tube was mounted onto the UV sensor by a plastic cylinder with an opening for visual control of tube filling (Figure S6, A – UV light off, B – UV light on). All experiments were conducted with necessary protection measurements (safety UV glasses and face shield, gloves, etc.)

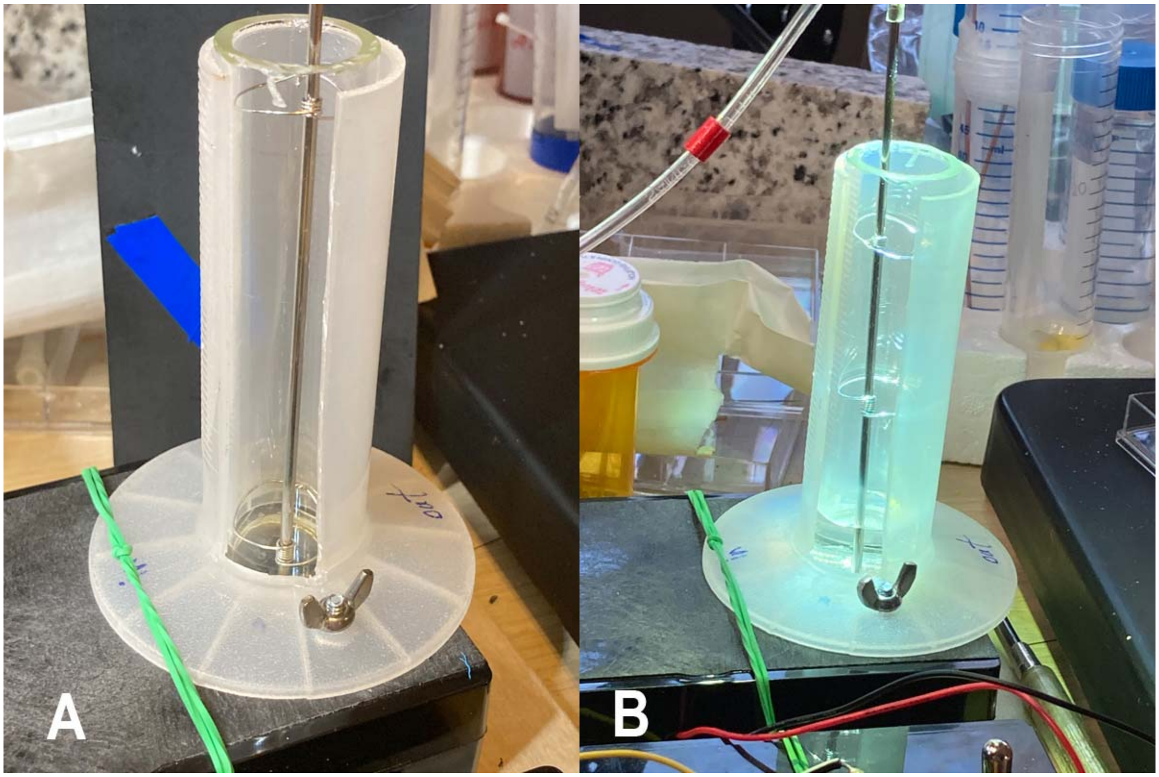

Figure S6.

Measurements

Before each measurement, the mercury bulb was warmed up for five minutes until the voltmeter reading stabilized at 3.67 V.

All stock solutions of salts were made as 2.5 g/L and then incrementally diluted with a step of 10%.
